# Awareness of intratumoral bacteria and their potential application in cancer treatment

**DOI:** 10.1007/s12672-023-00670-x

**Published:** 2023-05-06

**Authors:** Yin Liang, Qiyan Li, Yulin Liu, Yajie Guo, Qingjiao Li

**Affiliations:** 1 Department of Laboratory Medicine, The Eighth Affiliated Hospital, Sun Yat-Sen University, Shenzhen, 518033 China; 2 Department of Emergency, The Eighth Affiliated Hospital, Sun Yat-Sen University, Shenzhen, 518033 China

**Keywords:** Intratumoral bacteria, cancer treatment, Microbiome, Microbial community heterogeneity, Omics technology

## Abstract

Hitherto, the recognition of the microbiota role in tumorigenesis and clinical studies mostly focused on the intestinal flora. In contrast to the gut microbiome, microorganisms resident in tumor tissue are in close contact with cancer cells and therefore have the potential to have similar or even different functional patterns to the gut flora. Some investigations have shown intratumoral bacteria, which might come from commensal microbiota in mucosal areas including the gastrointestinal tract and oral cavity, or from nearby normal tissues. The existence, origin, and interactions of intratumoral bacteria with the tumor microenvironment all contribute to intratumoral microorganism heterogeneity. Intratumoral bacteria have a significant role in tumor formation. They can contribute to cancer at the genetic level by secreting poisons that directly damage DNA and also intimately related to immune system response at the systemic level. Intratumoral bacteria have an impact on chemotherapy and immunotherapy in cancer. Importantly, various properties of bacteria such as targeting and ease of modification make them powerful candidates for precision therapy, and combining microbial therapies with other therapies is expected to improve the effectiveness of cancer treatment. In this review, we mainly described the heterogeneity and potential sources of intratumoral bacteria, overviewed the important mechanisms by which they were involved in tumor progression, and summarized their potential value in oncology therapy. At last, we highlight the problems of research in this field, and look forward to a new wave of studies using the various applications of intratumoral microorganisms in cancer therapy.

## Introduction

Tumors are new organisms formed by overgrowth and abnormal differentiation of normal cells in the organism, which are not required by the body under the long-term action of different initiating and promoting factors. The normal cells acquire many abilities on their way to tumor-growing state, known as the hallmarks of cancer. Among them we note the polymorphic microbiomes, defined as “enabling hallmarks”, are the driving factors of tumor development and other signature features [1]. The research on tumors and microorganisms dates back to the nineteenth century, when attention was focused on the application of microorganisms in the tumor treatment. And at that time “a universal bacterial origin of cancer” was proposed, but later rejected because there was no mechanistic evidence to explain the reproducible results [2]. Fortunately, tumor microbiology has made advanced in recent years, and the field upholds the importance of microorganisms (both bacterial and fungal) in cancer and cancer treatment. 11 species of microorganisms have been identified as carcinogenic, many of which appear to be complicit in the growth of cancer [3]. Microorganisms live in the mucosal areas of the body that are connected to the outside world, and their changes are associated with the risk of cancer [4]. For example, the carriage of oral pathogens (*Porphyromonas gingivalis* and *Aggregatibacter actinomycetemcomitans*) were strongly associated with a higher risk of pancreatic cancer, while Fusobacteria, Leptotrichia genus were correlated with a reduced risk of pancreatic cancer [5]. Various experimental studies have also reported tumor-promoting functions of bacteria, such as the ability of Fusobacterium nucleatum to promote the development of colorectal cancer [6].

**Fig. 1:**
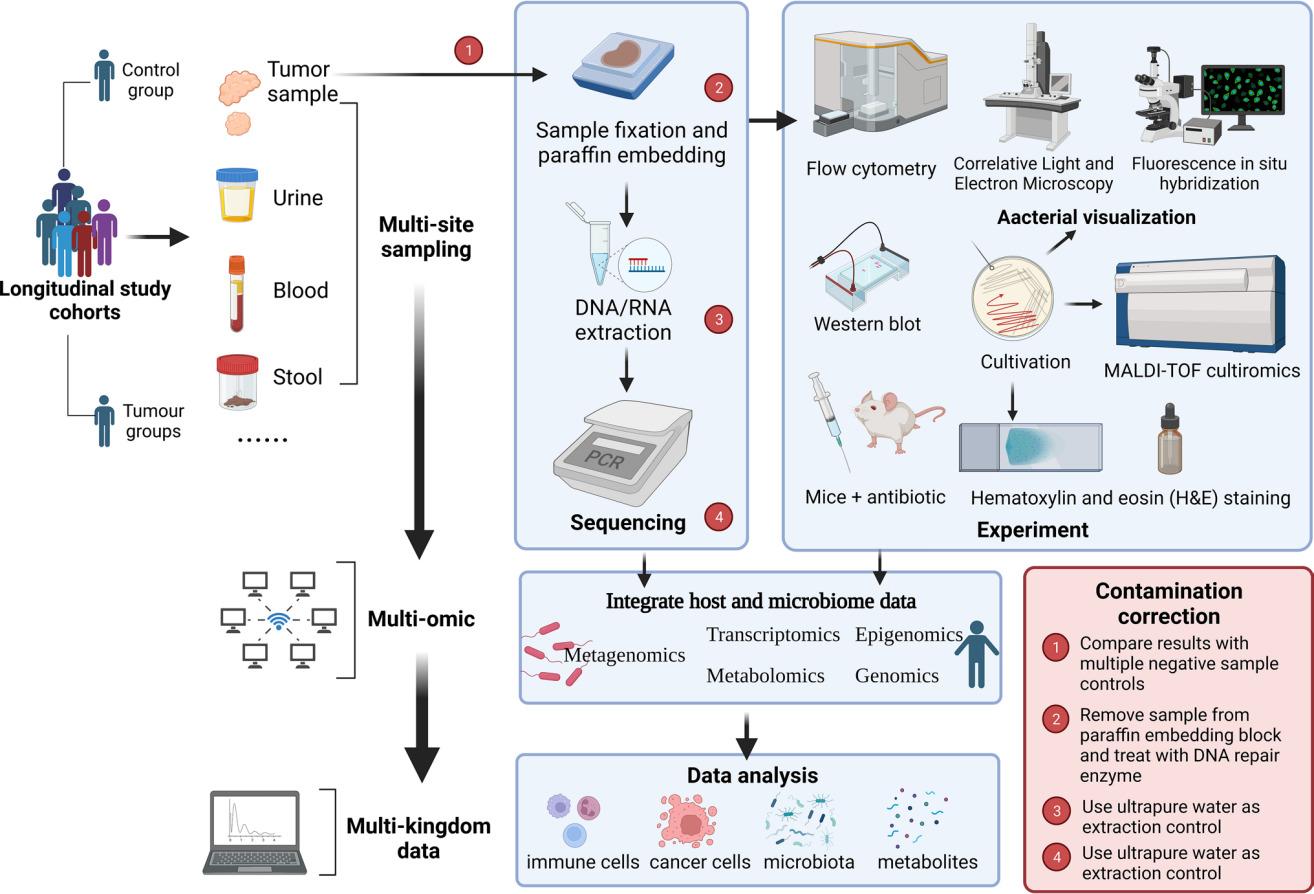
Multi-omic study strategy of microorganisms within tumors. Blood, stool, and tumor tissue samples obtained from longitudinal study cohorts are subjected to DNA or RNA extraction to gather host and microbial sequence data by different sequencing platforms and means. Smear and fluorescence staining may confirm the presence of microbes, while animal tests can confirm their function, MALDI-TOF cultiromics can identify microorganisms, and western blot can offer proteomics data. Combined multi-omics analysis techniques generate data from different domains, which in turn allow better study of host–microbiota interactions and explain the diversity of human microbe. On the other hand, from collecting specimens to performing gene sequence assays and then analyzing the data, there is a possibility of various contaminations. In the figure we propose corresponding contamination correction measures for the steps that are prone to contamination during pro-processing (created with BioRender.com)

Gut flora is associated with a variety of human diseases include cancer but the advent of second-generation sequencing technology has allowed the detection of microorganisms present in trace amounts in tumor tissue. The pancreas, for example, has long been thought to be sterile, but bacterial DNA was found in 76% of surgically removed pancreatic ductal adenocarcinoma (PDAC) samples and 15% of normal pancreatic control [7]. Multiple similar studies have demonstrated that microorganisms are present within different types of tumors and these intratumoral microorganisms are heterogeneous. Researchers have proven the involvement of microbiota in tumor development and the creation of local and systemic immune responses by working with sterile humanized mice as biomicrobial models [8, 9]. The vast majority of microorganisms use nucleic acid (DNA or RNA) as their genetic material, and the genes of microorganisms also have their specific species characteristics. Therefore, the detection and analysis of nucleic acid in samples has gradually become a convenient means of microbial detection, which is also a common method to detect intratumoral microorganisms [10]. In addition to microgenomics, which is dedicated to the study of microorganisms, there are also other omics techniques such as proteomics, transcriptomics and metabolomics. We are able to obtain multiple aspects of patient information through combined multi-omics analysis (Fig. 1) to better understand host–microbiota interactions, explain human microbial diversity, provide more evidence for the mechanism of microbial action in tumors, and explore candidate key factors at a deeper level. So that we can gain an in-depth understanding of the molecular mechanisms and genetic basis of complex traits in microbial and disease processes.

In this review, we mainly put the spotlight on the presence of bacteria within tumors and their mechanisms in regulating tumor development. We also outline the value of these microorganisms of interest in cancer therapy and explore the limitations of the research process.

## Awareness of microorganisms within the tumor

Microbiome is a newly discovered component of the tumor microenvironment (TME) [11]. The term “tumor microbe microenvironment (TIME)” refers to a milieu of microorganisms and their products within tumor tissue [12]. A large study characterizing the microbiome of 1526 samples from seven human tumor types, including breast, lung, ovarian, pancreatic, melanoma, bone, and brain, found that intratumoral bacteria are predominantly intracellular, present in cancer cells with immune cells [13]. Similarly, Huang et al. found enrichment of HCC microbial DNA in the cytoplasm of hepatocytes and erythrocytes by FISH, repeated hematoxylin and eosin (H&E) staining in serial sections and Gram staining [14].

**Fig. 2:**
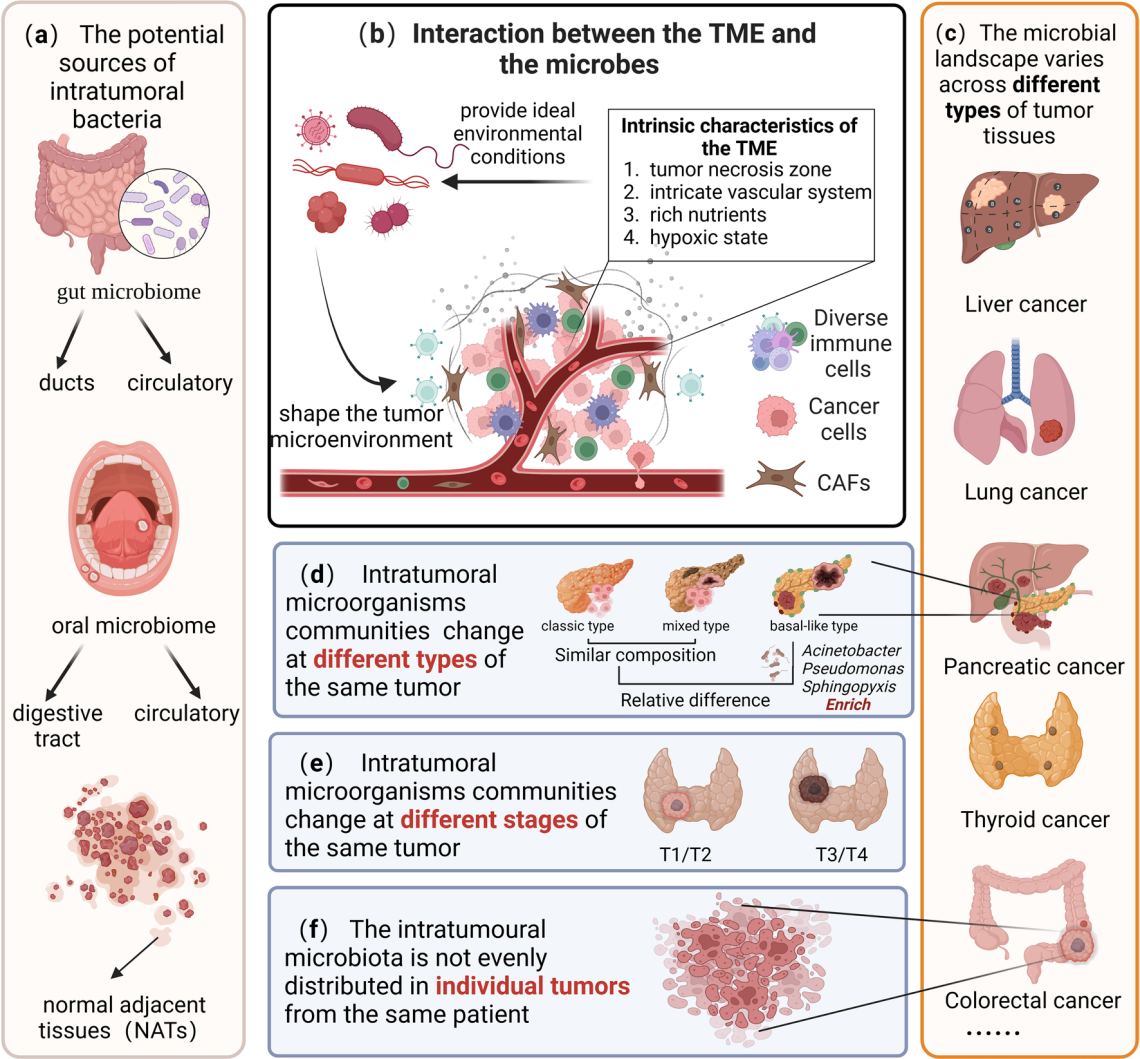
Heterogeneity of microorganisms within the tumor and potential sources. **a** Potential sources of intratumoral bacteria; **b** coexistence of TME and intratumoral bacteria; **c**–**f** heterogeneity of intratumoral microorganisms. In Figure **d**, classic and mixed pancreatic cancer tumors had similar microbiome compositions at the genus level, whereas basal-like tumors had a considerably distinct microbiome profile. The base-like subtype was significantly abundant in *Acinetobacter*, *Pseudomonas*, and *Sphingopyxis* (created with BioRender.com)

One of the potential sources of intratumoral bacteria is the mucosal tissues in the body that are connected to the outside world, and these sites are colonized with abundant commensal microorganisms, such as the intestine and oral cavity (Fig. 2a) [15]. Bacterial communities found in the tumor environment are mostly detected in the gut microbiome, suggesting that bacteria may be transferred from the gut to other parts of the tumor [16]. Pushalkar et al. demonstrated bacterial migration from the intestine to the pancreas by using a fluorescently labeled rat model [17]. Likewise, overlapping bacteria were also found in colorectal cancer tissues and paired metastatic liver tumor sites [18]. A separate report showed that the four major bacterial phyla in thyroid cancer were also major strains in the gut. When researchers set the threshold for abundance above 0.01, the papillary thyroid carcinoma (PTC) intratumoral microbiome was found to have different dominant strains compared to the gut microbiota at the genus level, but bacterial transferring from the gut to PTC still needs further exploration [19]. Oral microbiota is also one of the potential sources of intratumoral bacteria, Estemalik et al. found an overlap in the microbial composition of oral and prostate tumor sites, such as *P. gingivalis*, *Treponema denticola*, and *E. coli* [20]. *F. nucleatum* is a core resident member of the human oral microbiome and is rarely found in the intestine, where it is commonly thought to reach colon cancer tissue via the gastrointestinal (GI) tract [21]. A study found that intravenously injected *F. nucleatum* colonizes mouse tumor tissue in a Fap2-dependent manner, suggesting that *F. nucleatum* used a hematogenous pathway to reach colon adenocarcinoma. The results also showed that a host factor (Gal-GalNAc) and a microbial lectin (Fap2) could mediate enrichment in human tissue, human and mouse CRC cell lines, preclinical in situ CRC models, and *Clostridium difficile* enrichment in human CRC metastases [22]. But transient bacteremia could also enable *Clostridium oralis* to enter the circulatory system, making it hypothesized that they may reach the colorectum via the hematogenous route rather than the gastrointestinal route. The findings suggested that bacteria in prostate tumor tissue were likely to originate from the distal microbiota, but there was still considerable uncertainty about how these microorganisms reach within the distal tumor tissue. Some intratumoral germs, however, are rare in mucosal tissue at the relevant sites. This implies that there are other sources of intratumoral bacteria. Some studies have suggested that intratumoral bacteria may originate from normal adjacent tissues (NATs) to the tumor, because the bacterial composition of tumor tissues has a high similarity to NATs [13]. Even so, the causal link is not apparent whether the changes in the microorganisms in the tumor tissue influence the surrounding normal tissue or whether the cancer pushes the microorganisms in the surrounding normal tissue to infiltrate the tumor tissue.

It is sure that a consensus can be reached that tumor tissue and intratumor microorganisms are coexisting (Fig. 2b). On one hand, the intrinsic properties of tumor tissue provide ideal environmental conditions for microbial invasion and facilitate the accumulation of specific species of bacteria in the tumor. Intratumoral bacteria evade the immune system by manipulating the tumor necrosis zone as a natural immune barrier. The intricate vascular system and rich nutrients of tumor tissues are conducive to the colonization of tumor sites by microorganisms that spread with blood circulation. The hypoxic state of tumor sites is conducive to the reproduction of certain flora [23]. On the other hand, microorganisms can shape the TME systemically and locally. The gut microbiota and its products can enter the circulation to become part of the TME and regulate tumor growth in the distal tumor, while local microorganisms also have a shaping effect on the TME [13].

The diversity of microorganisms within the tumor is shaped by the existence of native microorganisms in diverse tissue locations, the number of commensal microorganisms in mucosal sites that can access the tumor site via multiple pathways, and the interaction between TME and microorganisms (Fig. 2). The microbial landscape varies across different types of tumor tissues (Fig. 2c), and Table 1 lists the alterations in microbial communities found in several recent intratumoral microbial investigations across malignancies. Riquelme et al. used multiple methods to provide compelling data showing increased α-diversity in the microbiome characteristics of different pancreatic tumor tissue samples; another study using primary lung tissue samples and The Cancer Genome Atlas (TCGA) validation cohort illustrates an overall increase in the microbiome of *Aspergillus* in lung cancer, while an increase in the abundance of acid polyploid (*Proteobacteria*) was specifically found in squamous cell carcinoma [24]. There may also be differences in the intratumoral microbial landscape of different subtypes of the same tumor (Fig. 2d). In a study of pancreatic cancer, Guo et al. performed microbial analysis of three subtypes of pancreatic cancer, namely classic, basal-like and mixed. They showed that the basal-like subtype had a unique microbial community, with PCoA analysis indicating that the basal-like subtype can be distinguished from the other two subtypes. And they also find the base-like subtype was significantly abundant in *Acinetobacter*, *Pseudomonas*, and *Sphingopyxis*, suggesting that they may affect the progression of tumors [25]. Intratumoral microbial communities also change at different stages of the same tumor (Fig. 2e). A study on the intratumoral microbiome of thyroid cancer found that patients with advanced lesions (T3/T4) had significantly higher tumor bacterial diversity than patients with less advanced lesions (T1/T2) [19]. Of noteworthy interest is the heterogeneity of intratumoral microorganisms in individual tumors of the same patient (Fig. 2f). Liu et al. discovered that the abundance of some colorectal cancer-associated pathogens (e.g. *Clostridium difficile*, *Clostridium* sp. and *Prevotella*) varied considerably in individual tumors and that intratumoral variation in the abundance of individual microorganisms may vary along the adenoma-carcinoma cell sequence [26]. Jorge et al. also explored intratumoral microbial heterogeneity and revealed how the intratumoral microbiota contributes to tumor heterogeneity through a series of novel techniques. The authors first sequenced 16s rRNA from different sites of colorectal cancer patients’ tumors and observed that the intratumoral microbiota was inhomogeneously distributed in individual tumor tissues. Furthermore they confirmed the heterogeneous spatial distribution of these bacterial communities by RNAscope-fluorescence in situ hybridization (RNAscope-FISH) imaging [27].

**Table 1:** Heterogeneity of the microbial landscape within tumors

Cancer type	Microorganism	Change situation	Sample	References
Oral squamous cell carcinoma	*Fusobacteriaceae* (family) *Spirochaetaceae* (family) *Flavobacteriia* (family) *Fusobacterium* (genus) *Treponema* (genus) *Capnocytophaga* (genus)	Enriched	OSCC (n = 133)and healthy controls (n = 20)	[90]
	*Firmicutes* (phylum) *Actinobacteria* (phylum)	Decreased		
Esophageal cancer	*Bacteroidetes* (phylum) *Bacteroidaceae* (family) *Enterobacteriaceae* (family)	Enriched	Fecal samples from patients with EC (n = 15) and healthy controls (n = 10)	[91]
	*Firmicutes* (phylum) *Veillonellaceae* (family) *Prevotellaceae* (family)	Decreased		
	*Streptococcus* (genus) *Veillonella parvula* (genus) *Porphyromonas gingivalis* (species)	Enriched	ESCC patient (n = 156) and healthy controls (n = 18)	[92]
	*Streptococcus* (genus) *Bifidobacterium* *Subdoligranulum* *Blautia* *Rombauts* *Collinsella* *Paeniclostridium* *Dorea* *Atopobium* (genus)	Enriched	EC (n = 23) and healthy controls (n = 23)	[93]
	*Lachnospira* *Bacteroides* *Paraprevotella* *Butyricicoccus* *Tyzzerella* *Fusicatenibacter* *Sutterella*	Decreased		
	*F. nucleatum*	Enriched	EC (n = 325) and normal tissues	[94]
Liver cancer	*Pseudomonas* (genus family) *Rhizobiaceae* *Agrobacterium* (family)	Decreased	HCC (n = 11), iCCA (n = 8) and combined (n = 9)	[95]
	*Proteobacteria* (phylum) *Firmicutes* (phylum) *Actinobacteriota* (phylum) *Bacilli* (classes) *Actinobacteria* (classes)	Enriched	HCC (n = 64) and normal tissue (n = 28)	[14]
	*Gammaproteobacteria*	Enriched		
	*Bacteroides* Uncultured bacterium that belonged to *Lachnospiraceae* (family) *Romboutsia* (genus)	Enriched	Metastatic HCC (n = 19), and primary HCC (n = 62)	[96]
	*Bacteroides* *Lachnospiracea incertae sedis Clostridium XIVa*	Enriched	HBV-related HCC (n = 113) and healthy volunteers (n = 100)	[97]
	*Enterobacteriaceae* *Fusobacterium* *Neisseria*	Enriched	HCC and paracancerous tissue samples (n = 99)	[64]
	*Pseudomonas*	Decreased		
Colorectal cancer	*Fusobacterium* *Bacteroides* *Parvimonas* *Prevotella*	Enriched	CRC (n = 36) and adenoma (n = 32)	[26]
	*F. nucleatum*	Enriched	CRC (n = 12) and tumor-free liver tissue samples (n = 7)	[22]
Gastric cancer	*Helicobacter* *Lactobacillus* *Streptococcus* *Prevotella* *Bacteroides*	Enriched	GC (n = 520)	[98]
	*Oceanobacter* *Methylobacterium* *Syntrophomonas*	Enriched	GC (n = 53) and chronic gastritis (n = 30)	[99]
Pancreatic cancer	*Acinetobacter* *Pseudomonas* *Sphingopyxis*	Enriched	PDAC (n = 62)	[25]
	*Proteobacteria* (*Pseudoxanthomonas*) *Actinobacteria* (*Saccharopolyspora* and *Streptomyces*)	Enriched (LTS)	PDAC (n = 68)	[100]
	A. *ebreus* B. *Acinetobacter baumannii*	Enriched (male)	PDAC (n = 187)	[85]
	*Geobacillus kaustophilusHTA426* *Escherichia coli55989*	Enriched (female)		
	*Paracoccus* *Brevundimonas* *Prevotella* *Cutibacterium* *Streptococcus*	Enriched	PDAC (n = 18)	[7]
	*Bifidobacterium* *Fusobacteria* *Rothia*	Enriched	PDAC (n = 74), normal pancreas tissue (n = 134) and pancreatic cyst (n = 98)	[101]
Bladder cancer	*E. coli* *F. butyrate*-producing bacterium SM4/1 *G. oscillatoria*	Enriched	BLCA (n = 400)	[102]
Renal cancer	*Lactobacilliales *(order ) *Leuconostocaceae *(family )	Decreased	Renal carcinoma (n = 10)	[103]
Breast cancer	*Anaerobes*	Decreased	Normal breast tissue (n = 6), BRCA (n = 11) and lymphoid metastases (n = 4)	[104]
	*Facultative anaerobes*	Enriched		
	*Fusobacterium*	Enriched	BRCA (n = 50)	[55]
	*Proteobacteria*	Enriched	BRCA (n = 68)	[105]
	*Actinobacteria* *Propionibacterium acnes* (*Cutibacterium acnes*)	Decreased		
Lung cancer	*Acidovorax*	Enrich	Controls (n = 33) and LUNG (n = 143) and TCGA (n = 1112)	[106]
Prostate cancer	*Shewanella *(genus )	Enrich	PRAD (n = 102) and tissue sample ( n = 114)	[107]
	*Bacteriodes fragilis* *Staphylococcus saprophyticus* *Vibrio parahaemolyticus* *Saimiriine betaherpesvirus*	Decreased		
	*Lactobacillales *(order) *Streptococcaceae *(family) *Staphylococcus *(genus)	Decreased	PRAD (n = 16)	[108]
	*Staphylococcaceae *(family)	Enrich		
Melanoma	*Acinetobacter* *Actinomyces* *Comamonas* *Corynebacterium* *Enterobacter* *Roseomonas* *Streptococcus*	Enrich	Tumor-blood sample (n = 108)	[53]
Head and neck squamous cell carcinoma	*Firmicutes* (phylum) *Actinobacteria *(phylum) *Lactobacillales* (order) *Veillonellales* (order) *Neisseriales* (order) *Actinomycetales* (order ) *Streptococcus* (genus) *Veillonella* (genus) *Actinomyces* (genus) *Rothia* (genus)	Decreased	HNSC (n = 155) and normal sample (n = 22)	[109]
	*Fusobacteria *(phylum) *Spirochaetes *(phylum) *Fusobacteriales* (order ) *Spirochaetales* (order ) *Fusobacterium* (genus) *Treponema* (genus)	Enrich		

The abundance of microbes varies among different types of tumors. Based on the comparison of samples in the sample column, the changes of these microorganisms are divided into two states: enriched and decreased

## Mechanism of the role of intra-tumorigenic bacteria in tumor development

**Fig. 3:**
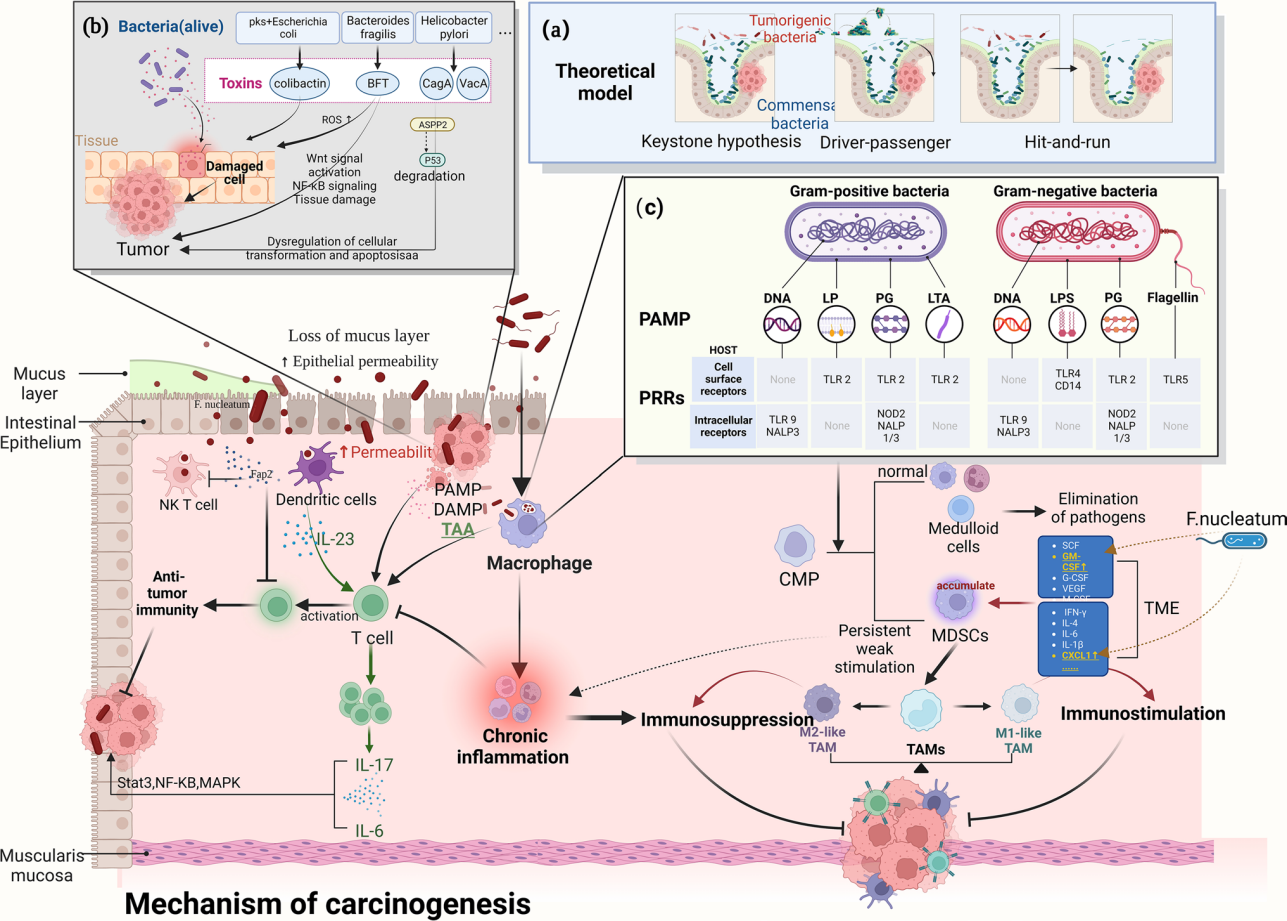
Carcinogenic mechanisms of microorganisms within tumors. Extensive involvement of the immune system in the pro-tumorigenic role of intratumoral microorganisms: The bottom left is about intratumoral microorganisms providing antigenicity and adjuvant properties, and he bottom right section is about the origin of TAM and its dual role in tumor progression. **a** Theoretical models for the involvement of intratumoral microorganisms in tumor development. It is notice that in the absence of other mutations, only the helicobacter pylori bacteria that cause cancer was identified as a key driver. Some other bacteria with carcinogenic properties have also received considerable attention, such as *Fusobacterium nucleatum*, *Escherichia coli* and *Bacteroides fragilis*, however further studies are needed to clarify the causal relationship between these bacteria and cancer; **b** intratumoral bacteria promote cancer development through toxin damage to DNA; **c** inflammatory inducers and associated sensor pattern recognition receptors that form chronic inflammation. *PAMP* pathogen-associated molecular pattern, *DAMP* damage-associated molecular pattern, *PRR* pattern recognition receptor, *TAA* tumor-associated antigen, *SCF* stem cell factor, *GM-CSF* granulocyte-macrophage colony-stimulating factor, *G-CSF* granulocyte colony-stimulating factor, *VEGF* vascular endothelial growth factor, *M-CSF* macrophage-stimulating factor, *CMP* common myeloid progenitor, *MDSCs* myeloid-derived suppressor cells, *TAMs* tumor-associated macrophages (created with BioRender.com)

The relationship and mechanisms between microorganisms and cancer have been well summarized from different perspectives in previous studies [4, 23, 28–31], but the mechanisms of the role of intratumoral bacteria in cancer development have been less elaborated. The current theoretical models of intratumoral microbial involvement in tumor development are showed in Fig. 3a. The presence of a single microorganism can cause tumorigenesis, named the “ladder hypothesis”; a single microorganism or a group of microorganisms that together with other microorganisms they recruit promote tumorigenesis is the “driver-passer” model; and the “hit-and-run” model has been described as transient colonization and injury by oncogenic bacteria, leading to tumorigenesis [32]. Regarding Keystone hypothesis, so far, in the absence of other mutations, only the helicobacter pylori bacteria that cause cancer was identified as a key driver. The driver–passenger model is easy to understand, and the damage or impact of one or a group of key cancer-driving bacteria on the surrounding environment during colonization and migration can recruit other interested bacteria to act together, or cooperate. This is consistent with the findings on microbes in tumors that not only a few bacteria differ, but the microbial composition of the whole tumor changes. The proposed “hit-and-run model” makes us aware that early microbial drivers or potential contributors may be lost when tumors are apparently forming, such as *Helicobacter pylori*, which has been identified as a class I carcinogen. Yet with the emergence of gastric tumors, the intragastric environment and TME are no longer suitable for *Helicobacter pylori* colonization, and the Poore GD study did not observe an enrichment of *Helicobacter pylori* in gastric tumors compared to adjacent normal tissue [33]. Here we present the mechanisms of microbial involvement in cancer development within tumors from both genetic and immune perspectives, and then relate these mechanisms to the stages of tumor progression.

### Intratumoral bacteria involving in cancer development at the genetic level through toxins

When not yet activated, proto-oncogenes do not have carcinogenic effects and maintain normal biological functions to control cell growth. Proto-oncogenes can be activated by alterations in gene structure (such as point mutations) or by alterations in the regulation of gene expression, which in turn result in the excessive or persistent presence of growth-stimulating signals that transform cells. Certain bacteria produce toxins capable of damaging DNA, leading to increased mutations that may eventually cause tumorigenesis, such as genotoxin-producing *Escherichia coli*, enterotoxin-producing *Bifidobacterium fragilis*, *Helicobacter pylori* [34], and ε and γ *Proteobacteria* [35]. Among the microorganisms and their toxins of interest in colorectal cancer-related studies, the Bacteroides fragilis toxin (BFT) produced by the *Enterotoxigenic Bacteroides fragilis* (ETBF) triggers E-calmodulin cleavage and bind to colonic epithelial cells (CEC) via unknown receptors, leading to increased barrier permeability, CEC Wnt signaling activation, c-Myc expression induction, and CEC proliferation amplification. Besides, BFT can regulate host gene expression and increase chromatin accessibility [36, 37]. BFT also generates high levels of reactive oxygen species (ROS), ultimately causes DNA damage and mutations [38]. Similarly, *E. coli* toxins produced by pks + *E. coli* have the toxicity to damage DNA and cause mutations [39]. Buti et al. found that the N-terminal end of the effector protein cytotoxin-associated gene A (CagA) encoded by type I *Helicobacter pylori* (Hp) strains interacted with the apoptosis-stimulating protein (ASPP2) of the tumor suppressor p53, resulting in proteasomal degradation of p53 and thus inhibiting the apoptotic response of the host cell [40]. Various signaling pathways are involved in the carcinogenesis of intratumoral bacteria, for instance, *Helicobacter pylori* in gastric cancer is the only bacterium currently identified as a carcinogen capable of encoding two toxins, cytotoxin-associated gene A (CagA) and vacuolar cytotoxin A (VacA) [4]. CagA is an important effector protein of Hp infection leading to inflammatory response and carcinogenesis in the host. CagA, which is typical of “oncoprotein”, can translocate from Hp into gastric epithelial cells and interfere with multiple signal transduction by activating extracellular signal-regulated kinase (ERK) signaling pathway, leading to malignant transformation of host cells. In contrast, VacA affects the β-linked protein signaling pathway and may play a role in the oncogenic potential of *Helicobacter pylori* [41].

### Extensive involvement of the immune system in the pro-tumorigenic effects of intratumoral microorganisms

Nearly 20% of human cancers and infections are associated with chronic inflammation [42, 43]. Common risk factors were known to be associated with cancer development during chronic inflammation, including *Helicobacter pylori* infection in gastric cancer, hepatitis B or C infection in liver cancer, and *human papillomavirus* (HPV) infection in cervical cancer [44–46]. The pathway of inflammation consists of four elements: inflammatory inducers, cellular sensors, inflammatory mediators, and the tissues and organs on which they act. Exogenous pathogen-associated molecular patterns (PAMP) and endogenous damage-associated molecular patterns (DAMP) are two types of inflammation inducers. Innate immune cells recognize these inducers via sensor pattern recognition receptors (PRRs), resulting in an acute inflammatory response for defense. This step is primarily recruited by neutrophils, and when acute inflammation is insufficient to resist, adaptive immunity by T cells and B cells takes over [47].

Toll-like receptors (TLR) and Nod-like receptors (NLR) are two important types of sensors in innate immunity. They combined with different structures of intratumoral bacteria and activated multiple downstream intracellular signaling pathways, leading to the release of excessive pro-inflammatory mediators. As during ecological dysregulation, immune activation via TLR and NLR signaling causes low levels of colonic inflammation, a known common factor in all colorectal cancers (Fig. 3c) [48]. Flagella of bacteria in the skin microbiota can influence skin cancer progression by producing a pro-inflammatory response through TLR5 receptor-mediated signaling [49]. Normally, strong stimulation of TLRs and PRRs can activate the classical pathway of common myeloid progenitor (CMP) differentiation to form mature myeloid cells and thus eliminate pathogens. But in the presence of continuous stimulation of chronic inflammation (such as tumors), the activation pathway of myeloid cells is altered to form immature myeloid-derived suppressor cells (MDSCs). MDSCs differentiate into tumor-associated macrophages (TAMs) after migrating to the tumor foci. TAMs are capable of being induced to undergo polarization into M1-type TAMs and M2-type TAMs in the organism, and the balance between M1 and M2 results in TAMs that sometimes promote cancer clearance and sometimes accelerate cancer progression and drug resistance in patients (Fig. 3) [50]. Udayasuryan et al. found that *F. nucleatum* infection increased the secretion of granulocyte-macrophage colony-stimulating factor (GM-CSF), CXCL1, IL-8, and macrophage inflammatory protein 3α, which could contribute to the accumulation of MDSCs in the TMA. It could be that GM-CSF promotes the proliferation of pancreatic cancer cells [2, 51]. In addition, the inflammasome PRR is involved in the formation of a protein complex that activates inflammatory pathways through the recognition of PAMPs or DAMPs by associated receptors, and inflammasome defects cause inflammation-induced colorectal cancer [4]. *Streptococcus lysosus* in colon cancer induces a pro-inflammatory state characterized by high NFκB and IL8 mRNA tissue expression [52].

Intratumoral bacteria provide antigenicity and adjuvant properties that foster an inflammatory tumor immune microenvironment. Tumor-associated antigen (TAA) can be presented via human leukocyte antigen (HLA) molecules on tumor cells and tumor-infiltrating immune cells to trigger anti-tumor responses. Certain microbial antigens have similar antigenic epitopes to tumor antigens and recruit microbial-specific T cells that can recognize and kill tumor cells [12, 53]. Bacteria within tumors can directly suppress anti-tumor immunity by inhibiting cytotoxic immune cell infiltration and blocking their ability to kill tumor cells. For example, Fap2 expressed by *F. nucleatum* in colon cancer directly binds to TIGIT receptors expressed on natural killer (NK) cells and T cells, inhibiting the antitumor activity of NK cells and T cells (Fig. 3) [54]. *F. nucleatum* colonizes in situ breast cancer and inhibits tumor-infiltrating T cells to promote tumor growth and metastasis [55]. Intratumoral bacteria also enhance infiltration and differentiation of immunosuppressive cells to promote tumor progression. For example, *Clostridium perfringens* induces immunosuppressive myeloid-derived suppressor cells, which can interfere with immune surveillance [54]. The immunological response mediated by helper T cell 17 (Th17) is normally designed to safeguard the body. When epithelial barrier deficiencies and mucin production problems arise in a malignant condition, intratumoral dendritic cells catch microbes and create IL-23, whereas other cell types produce IL-6 and IL-1. T cells are recruited and generate IL-17 in response to the actions of IL-23 and IL-6. Epithelial cells identify IL-17 and IL-6 and use Stat3, NF-KB, MAPK, and other signaling cascade events to promote cancer [39].

### The association between oncogenic mechanisms of intratumoral bacteria and the stages of tumor progression

Carcinogenesis can be split into three stages: the initiation stage, the promotion stage and the progression stage [56]. Intratumor bacteria are involved in all stages of tumor progression and can affect cancer cells from both external interactions and intracellularly. We define uncontrolled cell growth caused by spontaneous or induced genetic changes as the initial stage of tumors. For instance, the body exposure to certain carcinogens that alter the responsiveness of cells to the environment to provide a proliferative advantage for cancerous cells, and some bacterial toxins through bad DNA causes genetic mutations, etc. In addition *Fusarium foetida* is able to adhere to oral mucosal tissues, induce host organism immune response, and cause cyclooxygenase 2 (COX-2) overexpression, thus inhibiting programmed tumor cell death and eventually allowing tumor cells to continue to divide [57]. The tumor-promoting phase is a period of proliferation and accumulation of preneoplastic cells, inducing additional genetic damage and amplifying mutations. In esophageal cancer, LPS induces the body to produce pro-inflammatory cytokines, tumor necrosis factor and activate TLR4. They directly or indirectly activate NF-κB signaling pathway, leading to a vicious cycle of esophageal microflora dysbiosis and inflammatory response to promote tumorigenesis. In the study of colon cancer, ETBF-produced BFT impairs colonic epithelium and barrier integrity leading to oncogenic Th17-dominated inflammation, which induces epithelial cell proliferation through activation of NF-KB, STA3 signaling pathways and promotes cancer development [39]. Further tumor expansion and metastasis are characteristic of tumor progression. Since the efficiency of expansion and metastasis of cancer cells is very low due to environmental stresses, it is reasonably suspected that microorganisms residing in tumor tissue and present within tumor cells are involved in multiple steps of tumor metastasis [28]. Kong et al. reported that *Fusarium nucleatum* induced colorectal cancer by increasing the levels of CYP2J2 and 12, 13-EpOME through activation of TLR4/Keap1/NRF2 signaling, bringing about cause invasion and metastasis of colorectal cancer [58]. Another recent study on colorectal cancer metastasis found that *F. nucleatum* induced a novel pattern recognition receptor, ALPK1, to activate the NF-κB pathway, gave rise to upregulation of ICAM1, which in turn enhanced the adhesion of colorectal cancer cells to endothelial cells [59]. For breast cancer, *F. nucleatum* colonizes in situ breast cancer tissue and inhibits tumor-infiltrating T cells to promote tumor growth and metastasis [55]. In urinary tract-associated cancers, cytotoxic necrosis factor 1 (CNF1) secreted by *E. coli* affects a variety of cellular processes (e.g. inflammation, survival, cell adhesion and motility) by modifying Rho GTPase activity and actin cytoskeletal arrangement activation of Ras homologue family member C (RhoC) in bladder cancer cells. Hypoxia-inducible factor 1 (HIF1) stability was regulated by CNF1-mediated RhoC activation to increase the secretion of VEGF to promote angiogenesis for migration and invasion of prostate cancer cells [60, 61]. It has also been recently shown that bacterially infected tumor cells may increase secretion of exosomes to promote tumor progression and metastasis [62]. Moreover, R. Domenis reported that intratumoral bacteria are likely to activate TLR4 by secreting exosomes to influence tumor progression, which means that cancer cells can mobilize tumor metastasis through paracrine exosomal signaling rather than direct invasion of bacteria [63]. Interestingly, in the metabolic pathways involved in HCC-associated microbes, lipid metabolism was enhanced, which generally occurs in rapidly proliferating cancer cells. This finding suggests that microbial metabolites in the TME may be a potential source of lipid metabolism in cancer cells to promote cancer cell proliferation and invasion [64].

## The application value of intratumoral bacteria in oncology treatment

Surgery, chemotherapy, and radiation therapy remain the mainstays of cancer treatment. Most of them are effective against tumors, but all have limitations to varying degrees. For example, surgery is often time-consuming and complicated. Chemotherapy and radiotherapy often fail to distinguish between healthy and malignant tissue. Although these treatments have a wide range of action and can quickly limit the progression of cancer, they lack the ability to target the tumor area, leading to incomplete clearance of cancer cells and potential cancer recurrence. The side effects of these treatments may further damage DNA in healthy tissues and cause additional cancer changes, as well as the persistent risk of drug resistance, all of which affect cancer mortality in various degrees [23]. The following section describes the impact of intratumoral bacteria on cancer chemotherapy, immunotherapy, radiotherapy and the value of intratumoral bacteria in targeted therapy.

### Association of intratumoral bacteria with cancer chemotherapy resistance

**Fig. 4:**
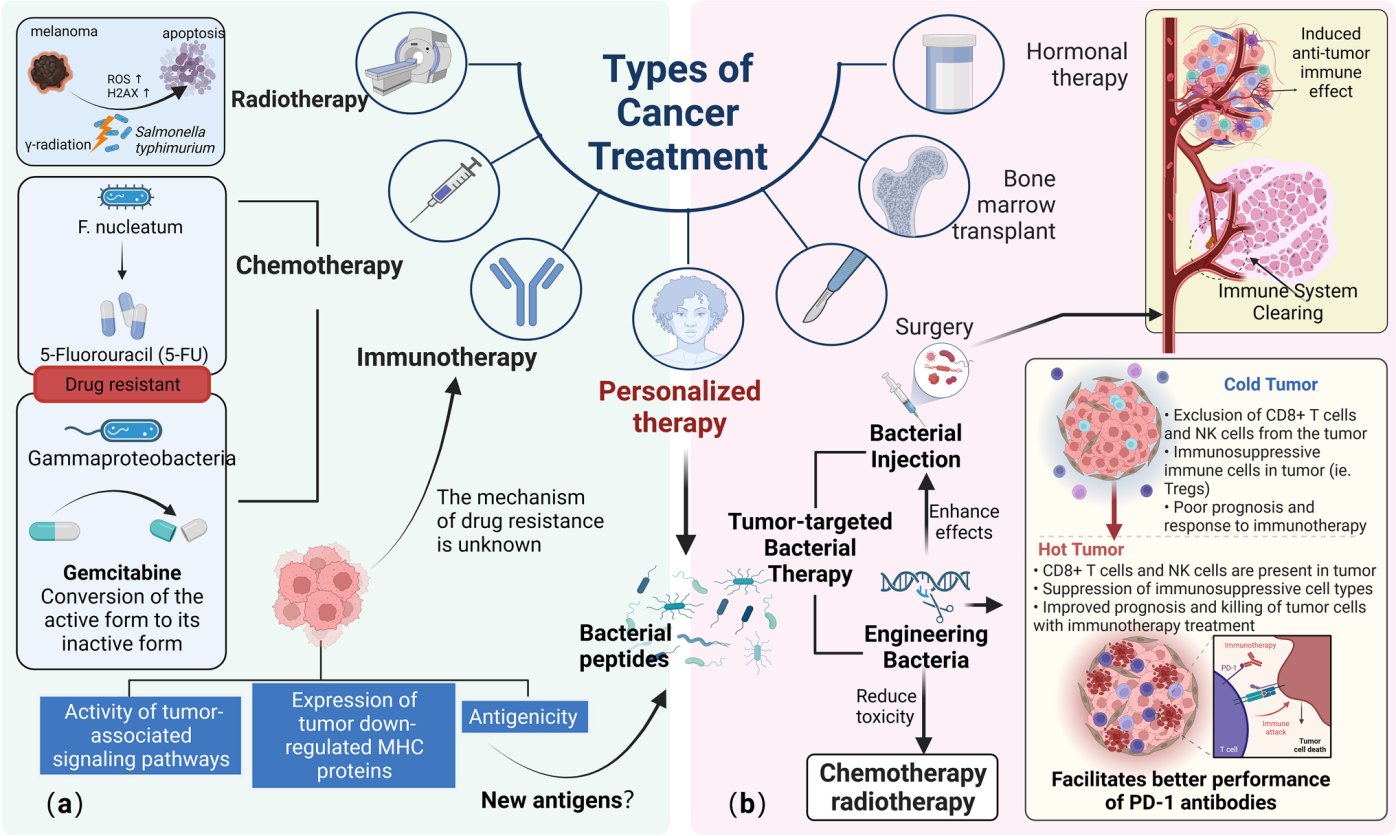
The value of intratumoral bacteria in oncology treatment. **a** Effect of intratumoral bacteria on chemotherapy, radiotherapy and immunotherapy; **b** combination of bacteriotherapy and cancer treatment (created with BioRender.com)

Tumor drug resistance is a major problem limiting the efficacy of current cancer chemotherapy drugs. Many cancer patients have remarkable efficacy in the early stage of chemotherapy, but with longer treatment time, the drug resistance of cancer cells increases, eventually leads to treatment failure [65]. The mechanisms of chemical drug resistance involve genetic alterations, DNA damage repair, epigenetic modifications, dysregulation of apoptosis, autophagy and changes in the TME [66, 67]. Tumor tissues are rich in microorganisms, which are involved in tumor progression in various ways and likewise have an impact on the effectiveness of chemotherapy. Yu et al. found that colorectal cancer tissues from patients with recurrence after chemotherapy were abundant in the intestinal bacterium *F. nucleatum.* These bacteria are able to modulate specific microRNAs through TLR4 and MYD88, activate autophagy pathways, alter colorectal cancer chemotherapy responses, and ultimately contributing to 5-fluorouracil (5-FU ) resistance [68]. Similarly, Geller et al. discovered an active form of gemcitabine (2′,2′-difluorodeoxycytidine) could be converted to its inactive form (2′,2′-difluorodeoxyuridine) by *Gammaproteobacteria* expressing the bacterial enzyme cytidine deaminase (CDD_L_) in a colon cancer model (Fig. 4a) [69]. In some non-gastrointestinal cancers, local bacteria have been found to potentially act as in situ biotransformation reservoirs that may complicate cancer therapy by reducing antitumor efficacy or increasing off-target toxicity [70]. In addition, Geller et al. found that most of the bacteria inside pancreatic tumors belonged to the *γ-Amastigotes* phylum, and they all had the CDD_L_ enzyme that “digests” gemcitabine. When strains isolated from human pancreatic tumors were cultured separately with cancer cells, most of them became responsible for helping cancer cells to become resistant to gemcitabine. And this demonstrates that such gemcitabine-depleting microorganisms are also present in human pancreatic cancer tumors [69]. The above studies show that the use of antibiotics can relieve the drug resistance of tumors, and the adjuvant treatment of antibiotics can be added to the treatment of tumors to improve the effect of cancer treatments. Further, the direct use of antibiotics cannot achieve targeting, that is, it does not affect other beneficial microorganisms, so the development of drugs against CDD_L_ may be a better choice.

### The impact of intratumoral bacteria on immunotherapy

In comparison to chemotherapy and radiotherapy, antitumor immunotherapy does not kill tumor cells directly through cytotoxic treatments and chemotherapeutic agents, but rather enhances the host’s immune system to recognize and target tumor cells [71]. Immune checkpoint inhibitors (PD-1/CTLA4), for example, help to restore anti-cancer activity and represent an emerging trend in immunotherapeutic approaches, but unfortunately this therapy only works in about 25% of patients [72]. Intratumoral microorganisms play a non-negligible role in antitumor immunotherapy, and one study reported significant differences in the intratumoral bacterial taxa of melanoma patients who responded to immune checkpoint inhibitors compared to those who did not respond [13]. In turn Shi et al. also found that systemic administration of *bifidobacteria* led to their accumulation within the tumor and enhanced the response to anti-CD47 immunotherapy in an interferon gene (STING) and interferon-dependent manner [73]. Recent studies have shown that cancer cells can also be resistant to immunotherapeutic drugs, though the mechanism of this resistance is still obscure. The ability of tumors to resist immunotherapy is related to a number of factors, of which the most important is the tumor cells themselves, including the antigenicity of tumor cells, the expression of tumor down-regulated MHC proteins, and the activity of tumor-associated signaling pathways (Fig. 4a) [74]. Tumor cells with lower antigenicity are more resistant to immunotherapy, while tumor neoantigens, which distinguish tumor cells from normal cells, arise mainly from genetic mutations. For example, people with more mutations, such as melanoma, have a higher response rate to immunotherapy. There is growing evidence that neoantigens are pivotal cancer immunogens, as a recent study by Kalaora suggests that bacterial peptides presented by tumor cells may be a previously unidentified tumor antigen [53]. They found bacteria colonized within melanoma can enter melanoma cells and their peptides can be presented by HLA-I and HLA-II molecules in melanoma. Since both classes of HLA molecules play a central role in CD8^+^ and CD4^+^ T cell immunity, they are expected to ultimately regulate immune function. It is noteworthy that the presence of bacterial peptides in normal tissues is unknown, and the number of identified melanoma neoepitopes is quite abundant, yet they do not elicit an effective human response to melanoma. Further studies of tumor-displayed bacterial peptides in combination with patient information may help researchers to select appropriate bacterial targets for cancer immunotherapy approaches [53].

### Cooperation between tumor microbiota and radiotherapy

Radiotherapy is a kind of local treatment of tumor using radiation. Radiation can damage the DNA structure of tumor cells, thus inhibiting tumor cells growth to achieve the effect of remission. In fact, as with chemotherapy, the efficacy of radiotherapy depends in part on inducing immunogenic cell death. Shiao et al. showed that the fungal microbiota, which together with the bacterial microbiota shapes radiation-induced therapeutic antitumor immunity, can control the immune response to cytotoxic therapy [75]. The management of intestinal flora in radiotherapy is the subject of numerous studies right now, and it has been discovered that intestinal flora can improve radiotherapy’s effectiveness and lessen its negative effects. On the other hand, *Salmonella* is used as a vector system in melanoma cells for systemic delivery of cytokine treatments such as oral cytokine gene therapy. Yoon et al. found that attenuated *Salmonella typhimurium* combined with gamma radiation increased ROS level and H2AX phosphorylation levels, thereby inducing apoptosis by caspase-3 and bcl2 in tumor cells (Fig. 4a) [76]. In the future, microorganisms might be used to improve radiation treatment.

### Tumor-targeted bacterial therapy

The ideal cancer treatment should preferably be able to target any specific tumor tissue, respond appropriately to external stimuli, and adjust properly to the body’s environment or even become self-sufficient within the body [77]. The key to bacterial therapy is that the bacteria must possess high targeting ability, low toxicity, or act as live delivery vehicles to penetrate into tumors with cytokines, cytotoxic factors, regulatory factors, prodrug invertase and small interference RNA (siRNA) to act on potential targets. Two modalities are available for bacterial cancer therapy, one is intravenous infusion of live bacteria and the other is intratumoral injection of inactivated bacteria. The nature of the TME provides a unique and preferential microenvironment for the colonization of these bacteria, which are targeted for transporting into solid tumors by various mechanisms. Simultaneous systemic injections result in the spread of live bacteria to normal tissues as well, but these live bacteria in the circulation and normal tissues are subsequently cleared by the organism (Fig. 4a) [78]. For example, the parthenogenic anaerobic bacterium *Salmonella* can be cleared by normal tissues within a few days after systemic administration with the help of immune function, while the immunosuppressive microenvironment of tumors contributes to the colonization of the bacterium; individual bacteria also have their own specific tumor targeting mechanisms, like *Listeria monocytogenes* can infect not only antigen-presenting cells (APCs) but MDSCs, which are then transported to the TME through MDSCs [79]. Bacteria arrive in solid tumors and exert their antitumor effects through different mechanisms, some common and some unique, similar to the above mentioned mechanisms of microbial involvement in tumor progression within the tumor. Both live and inactivated bacteria can induce intrinsic and adaptive immunity in the body, thus altering the tumor immune microenvironment to exert antitumor effects.

With the continued development of genetic technology and synthetic biology, it is possible to modify bacteria to enhance their safety and antitumor activity. Attenuated bacterial therapy improves safety by deleting the major virulence genes of bacteria or by cultivating nutrient-deficient mutant strains. Attenuated bacteria accumulate specifically in tumors and can directly induce apoptosis and autophagy and inhibit tumor angiogenesis in tumor cells. Moreover, attenuated bacteria can enhance the host immune response to tumors, including increasing and activating cytotoxic T lymphocytes and NK cells, reducing tumor infiltration of Treg cells and ablating the immunosuppressive capacity of MDSCs. For example, as a result of engineering *Salmonella typhimurium* VNP20009 increased cysteinase-3 activity and Bax protein expression to induce apoptosis of cancer cells in vivo and in vitro, thereby inhibiting pancreatic cancer growth [80]. Gene engineered bacteria can also transfer from the immunosuppressive microenvironment by modulating innate and adaptive immune responses, inducing immunogenicity and ultimately improving the host’s anti-tumor immune response. Bacteria can be made to express ligands for certain tumor cell overexpression molecules by engineering them to express antibodies against tumor-associated antigens or by using special gene promoters to make proteins or peptides expressable only at the tumor [78].

Modified according to the corresponding needs, engineered bacteria can also act as vectors to penetrate deep into the necrotic and hypoxic regions of tumor tissues to express cytokines, anticancer drugs, precursor drug-converting enzymes, immunomodulators in the form of DNA/RNA/proteins, and exert intrinsic tumor-killing and immune-stimulating activities [81]. Despite their apparent anti-cancer ability, genetically engineered bacteria cannot completely eradicate the growth or metastasis of cancer. It has a better anti-tumor effect when combined with other cancer treatments, such as combine checkpoint suppressor antibodies with the treatment of bacteria or viruses. The infiltration of T cells in the cold tumor microenvironment is poor. When bacteria are ingested into the body as a pathogen, they can break the silent immune microenvironment, activate immune cells to turn cold tumor into hot tumor. In addition, bacteria that produce and secrete checkpoint inhibitors can be directly targeted within tumor tissues, binding to and neutralizing T-cell inhibition sites expressed by tumor cells (Fig. 4) [78]. Another aspect is that the combination of chemotherapy with attenuated bacteria significantly improves anticancer efficacy and reduces toxicity (Fig. 4b) [82]. In addition, it is difficult for traditional gene expression control to achieve non-invasive, targeted and controlled induction of bacterial exogenous gene expression in tumors deep in vivo, limiting the application of engineered bacteria in tumor therapy. However, combined multidisciplinary approaches can facilitate the treatment of tumor immunity. Recently, a research team has developed a new method for immunotherapy of tumors based on focused ultrasound regulation of bacterial gene expression. Ultrasound-responsive engineered bacteria were obtained by designing an ultrasound-responsive gene expression circuit and introducing it into tumor-targeting bacteria. With the help of ultrasound’s excellent advantages of non-invasiveness, focusability, high tissue penetration and acoustothermal conversion, gene expression regulation of engineered bacteria can be realized at the tumor site. This system has been used to control the expression of interferon-γ (IFN-γ) by bacteria in tumors with ultrasound, and has been successfully applied to immunotherapy of subcutaneous transplanted breast cancer and in situ transplanted liver cancer with good results. It has great potential applications in the regulation of gene expression in living cells such as bacteria, immune cells and stem cells [83].

## Discussion

Exploring intratumoral microorganisms is intricate and challenging. The complexity lies in how to eliminate potential contamination and correct for the effects of contamination during the study, while the challenge is how to explore the causal relationship between tumors and intratumoral microorganisms, both of which are currently the focus of relevant studies.

A large number of related studies have shown that the abundance of microorganisms in tumors is very low, so any contamination during the detection and analysis process may affect the final results. In addition, the highly sensitive type of second-generation sequencing technology can detect low traces of microorganisms. At the same time, it also has a high detection rate for contaminated microorganisms in samples. How to identify true positive microbial signals in low-trace samples is the key to dealing with contamination [84]. Although reasonable and standardized laboratory operations can reduce environmental pollution, they cannot completely eliminate it. In the figure, we summarize the corresponding pollution correction measures for the steps that are prone to pollution in the processing process, and the core is to set a reasonable negative control (Fig. 1). Nejman et al. analyzed the microorganisms in seven human tumors by strictly controlling contamination. Because of the wide range of samples and types, the contamination treatment of the samples was very careful. A DNA-extraction control group, an amplification blank control group, a sequencing quality-control group, and a marginal paraffin sample control group (paraffin samples were used to account for an effect of the environment of the sectioned sample). Regarding samples, they selected resected samples of solid tumors from different experimental centers. In terms of filtering methods, the researchers built a RIDE check, that is, detailed documentation of the experimental design to reduce all possible contamination, detailed documentation of all information at the time of sequencing to assess possible contamination of DNA testing, determining and minimizing the impact of DNA contamination (how to reduce false positives), and understanding and reporting on the possible impact of contamination that cannot be removed [13]. Four data contamination correction schemes were proposed by Chakladar et al. for tumor RNA sequencing data obtained from public databases, comprising contamination assessment using sequencing dates, contamination assessment of base sequencing lanes, and contamination assessment using microbial abundance counts and contamination assessment using previously identified contaminants [85]. The engineers built some machine algorithms as well as R language packages to handle the contamination correction process in the downstream analysis, which greatly improved the research efficiency [86]. After these steps, more credible potential microorganisms were screened for further analysis. It should be noted that for sequencing data obtained directly from public databases or other studies, because the sequencing data of some studies are not specific to microorganisms in the study, the data related to microbial contamination are incomplete, such as the list of common contaminating microorganisms of the sequencing laboratory, the total DNA concentration of the sample, and the batch and specific time of sequencing. As a result, the interference cannot be excluded by the method proposed by Chakladar et al., which affects the final analysis results. This prompts us to record the relevant information as comprehensively as possible during the detection and analysis of samples, make full use of the effective information of each sample, and explore the biological significance behind the data.

That most studies of microorganisms associations in cancer to date have been cross-sectional, rather than prospective and longitudinal [87]. Gastric cancer and *Helicobacter pylori* currently represent the strongest link between a single bacterium and cancer causation [32]. *Helicobacter pylori* is the only microorganism identified as a carcinogen that is capable of being collected, transferred in a healthy host as disease replicates, and re-isolated from an inoculated host in accordance with Koch’s theorem, which satisfies a criterion in the causality corollary that elimination or control of the microbiota reduces the incidence of disease [87]. One of the reasons why most of the tumor-associated microorganisms that have received attention today have not been able to fully elucidate their causal relationship with tumorigenesis is the limitations of the methods used to explore causality. The rodent model is an important model for studying causality, but this model is likely to have restrictions such as inhomogeneous microbial colonization and lack of host disease state simulation in the course of the study [31]. In fact, how to obtain microbiological data from normal samples in compliance with ethical requirements is a major difficulty, and how to define normal controls in the study and obtain the appropriate specimens remains an important issue.

Fortunately, the advent of an increasing number of new technologies and analytical methods has facilitated advances in the study of intratumoral microorganisms. Spatial transcriptomics generates full transcriptomic data from a complete tissue sample, which in turn enables the localization and differentiation of active expression of functional genes within specific tissue regions, providing valuable insights for research and diagnosis. By adapting and applying these techniques, Jorge et al. developed a single-cell RNA sequencing technology, invasive adhesion directed expression sequencing (INVADEseq), which they applied to oral and colorectal cancer studies to identify cell-associated bacteria and the host cells they interact with and to reveal alterations in transcriptional pathways involved in inflammation, metastasis, cellular dormancy, and DNA repair [27]. This research has moved from studies of the relevance of microbial communities to cancer toward the analysis of the functional impact of microbial communities in tumors, allowing the identification of molecular and cellular targets for these cancers. In addition, a team of researchers from Harvard university and MIT recently invented microbe-seq, a high-throughput single-cell genomics technology for microorganisms, which can obtain genomic information of thousands of single-cell microorganisms from complex microbial communities without culture and assemble high-quality strain-level genomes. It has important theoretical and practical implications for discovering microbial resources with potential applications and exploring inter/intra-species relationships [88]. Likewise, Martin Blaser and Subhajyoti De at Rutgers university, and others have developed a single-cell analysis method, SAHM, capable of obtaining individual tumor cells and their associated microbial sequences by single-cell sequencing, and ultimately obtaining authentic and reliable microbial sequences by noise reduction processing, and thus studying host-microbe interactions. Based on the SAHMI approach, the authors found that microorganisms were indeed present in pancreatic tumor tissue and significantly affected gene expression and activated tumor immune responses in host cells [89]. But compared to cancer cells, the abundance of microbial cells within tumors is low, knowledge of their function and potential remains limited, and further validation of their prevalence and impact in different cohorts and therapeutic contexts is needed.

Bacteria have a natural ability to target tumors and can be easily engineered to enhance the anti-tumor ability and reduce the virulence of some therapeutic bacteria, such as the landmark BCG vaccine, depending on available needs. By using bacteria for treatment, the bacteria themselves not only have tumor lysis effect, but also can reactivate immune cells like activators. Additionally, the bacteria can be modified by genetic engineering to become intelligent carriers for drug delivery, so as to achieve deep drug delivery in the tumor, and thus hit the tumor target precisely. There are actually many limitations to the studies of engineered bacteria, such as clinical studies have not produced satisfactory results; the degree of attenuation of toxicity leads to differences in therapeutic efficacy, and if toxicity is not sufficiently attenuated, they may proliferate in the blood and even cause severe infectious shock but excessive attenuation of toxicity can also reduce the ability of bacteria to treat cancer; the ability of bacteria to accumulate in tumor tissue; balancing bacterial virulence and anti-tumor capacity; genetic instability, etc. Besides, we need to consider the bacterial species, the tumor type, and the stage of bacterial–host interaction when making specific modifications [81].

Intratumoral bacteria have an inestimable potential to distinguish normal and cancerous tissues, affect the degree of response to drugs and the prognosis of cancer treatment. Also the spatio-temporal heterogeneity of intratumoral bacteria is an important support for their use as drug carriers for precise targeting of therapy. In the future, we expect to discover more meaningful intratumoral microorganisms and explore the causal relationship between these microorganisms and tumor progression, so that we can apply them to cancer treatment to achieve precision treatment and reduce the side effects caused by the cancer treatment process.

## Data Availability

Not applicable.

## Abbreviations

EC: Esophageal cancer
ESCC: Esophageal squamous cell carcinoma
OSCC: Oral squamous cell carcinoma
PLCs: Primary liver tumors
iCCA: Intrahepatic cholangiocarcinoma
CRC: Colorectal cancer
GC: Gastric cancer
PDAC: Pancreatic adenocarcinoma
BLCA: Bladder cancer
BRCA: Breast cancer
LUNG: Lung cancer
PRAD: Prostate cancer
HNSC: Head and neck squamous cell carcinoma
